# Empagliflozin Alleviates Arsenic Trioxide–Induced Nephrotoxicity by Activating the SIRT1/Akt/Nrf2 Pathway

**DOI:** 10.1155/jt/5808911

**Published:** 2026-02-13

**Authors:** Chunrong Pang, Wenlei Zhang, Chenli Yue, Haoxuan Li, Jinyan Li, Xinru Wang, Xinsheng Duan, Longyu Li, Zengliang Gao, Xin Hai

**Affiliations:** ^1^ Department of Pharmacy, First Affiliated Hospital of Harbin Medical University, Harbin, Heilongjiang, China, hrbmu.edu.cn

**Keywords:** acute promyelocytic leukemia, arsenic trioxide, autophagy, empagliflozin, nephrotoxicity

## Abstract

Arsenic trioxide (ATO), utilized in the treatment of acute promyelocytic leukemia (APL), presents significant renal toxicity that restricts its clinical usage. The potential effects of empagliflozin (EMPA) on ATO‐induced nephrotoxicity remain unexplored. This study aims to investigate whether EMPA can alleviate ATO‐induced nephrotoxicity in both animal and cellular models, as well as to further explore the underlying mechanisms. EMPA can improve renal function in mice and alleviate ATO‐induced structural damage to the kidneys. EMPA treatment effectively inhibits ATO‐induced oxidative stress and reduces apoptosis. EMPA significantly decreases the production of ROS in cultured HEK293T cells, lowers the apoptotic rate, and safeguards mitochondrial function. EMPA upregulates the SIRT1/Akt/Nrf2 pathway and addresses ATO‐induced autophagy dysfunction. These findings suggest that EMPA may ameliorate ATO‐induced renal toxicity by activating the SIRT1/Akt/Nrf2 signaling pathway, which is associated with the suppression of oxidative stress, reduction of apoptosis, and protection of mitochondrial functionality.

## 1. Introduction

Arsenic is a toxic nonmetallic element that is widely distributed in the natural environment, posing a significant threat to human health [1]. The conventional approach to treating severe arsenic poisoning involves supportive strategies customized to the individual patient’s needs. This may include interventions like mechanical ventilation, fluid resuscitation, muscle relaxants, and gastrointestinal decontamination methods, such as gastric emptying and administering oral activated charcoal. Additionally, chelating agents are utilized to help decrease the arsenic concentrations in the body [2]. Despite its pronounced toxicity, arsenic trioxide (ATO) has been utilized clinically for many years, particularly as a targeted therapy for acute promyelocytic leukemia (APL) [3]. However, the application of ATO is constrained by dose‐dependent toxicity, with nephrotoxicity being especially notable [4]. Upon entering the human body, arsenic undergoes methylation predominantly in the liver, with 40%–60% of arsenic eliminated through urine [5, 6]. Arsenic may lead to kidney function impairment and disruption of the oxidative stress system. Decreased kidney function can hinder the body’s ability to effectively eliminate arsenic and its metabolites, potentially resulting in further damage. Research conducted by Wei‐Ying Jen et al. has demonstrated that high‐dose ATO treatment in APL patients is significantly associated with nephrotoxicity [7]. The underlying mechanisms of ATO‐induced renal toxicity remain incompletely elucidated; exploring new therapeutic strategies to reduce this nephrotoxicity remains an essential challenge.

The toxic effects of ATO on the kidneys are largely attributed to several interconnected mechanisms, including inflammation, oxidative stress, apoptosis, and autophagy. Exposure to arsenic can trigger oxidative stress in mice, resulting in a decrease in natural antioxidants like superoxide dismutase (SOD) and glutathione (GSH), while concurrently elevating the levels of malondialdehyde (MDA) and generating reactive oxygen species (ROS). These biochemical alterations ultimately culminate in cellular apoptosis, along with the subsequent release of proinflammatory factors that further exacerbate kidney damage [8, 9]. Studies demonstrate that ATO triggers apoptosis in NB4 cells by facilitating the breakdown of mitochondrial membrane potential (MMP) and causing mutations in mitochondrial DNA [10]. Mitochondrial impairment is considered a significant factor in renal damage [11]. The kidneys serve as the main excretory organs for arsenic and necessitate substantial amounts of energy and oxygen to support metabolic functions [12]. Dysfunction of mitochondria may be a significant factor in the development of kidney injury caused by ATO. While antioxidants such as curcumin and tannic acid have shown efficacy in alleviating ATO‐induced nephrotoxicity, no agents have been identified that effectively protect mitochondrial function [8, 9]. Searching for drugs that can enhance the endogenous antioxidant defense system in the kidneys and alleviate mitochondrial damage is a promising strategy for preventing ATO nephrotoxicity.

The Silent Information Regulator 1 (SIRT1) is essential for preserving mitochondrial function and managing levels of oxidative stress. It acts as a key modulator of cellular metabolism, affecting processes like energy generation, programmed cell death, and the cellular response to oxidative stress, ultimately aiding in the maintenance of overall cellular health [13]. Resveratrol, a SIRT1 activator, can activate SIRT1 and further activate the Akt pathway, inhibiting mitochondrial damage [14]. Nuclear factor erythroid 2–related factor 2 (Nrf2) is involved in safeguarding cells against damage caused by free radicals and preventing apoptosis by stimulating antioxidant enzymes while also inhibiting harm triggered by oxidative stress, thereby enhancing cell survival [15]. Protein kinase B (Akt), an essential player in cell survival, has demonstrated the ability to enhance Nrf2 expression and mitigate oxidative damage across different diseases [16]. Nrf2 functions as an essential transcription factor within the antioxidant defense mechanism. In conditions of oxidative stress, Nrf2 moves into the nucleus, where it attaches to antioxidant response elements, thereby enhancing the expression of downstream antioxidant genes, including NAD(P)H dehydrogenase quinone 1 (NQO1) [17]. Thus, we hypothesize that the activation of the SIRT1/Akt/Nrf2 signaling pathway could be effective in reducing ATO‐induced kidney damage.

Some traditional Chinese medicines and natural products can alleviate ATO‐induced nephrotoxicity in animal experiments, but the safety of these drugs in clinical applications still needs further verification [8, 9]. Empagliflozin (EMPA) is a sodium–glucose co‐transporter 2 (SGLT2) inhibitor that is widely used in clinical practice. It exhibits a range of pharmacological protective effects on renal and cardiac systems, including anti‐apoptotic, anti‐inflammatory, and antioxidant properties [18]. Furthermore, EMPA not only demonstrates antioxidant activity but also engages in various mechanisms of action through AMPK‐mediated autophagy pathways [19, 20]. EMPA is considered a potential SIRT1 agonist, with numerous studies indicating that it can upregulate SIRT1 expression [21].

In this investigation, we seek to assess whether EMPA can mitigate renal toxicity induced by ATO and examine its potential role in regulating the SIRT1/Akt/Nrf2 signaling pathway to diminish oxidative stress and improve mitochondrial function. The results of our research will offer new insights for the treatment of ATO‐induced kidney injury.

## 2. Materials and Methods

### 2.1. Chemicals and Reagents

ATO was purchased from Harbin YI‐DA Pharmaceutical Ltd (Harbin, China). EMPA, 3‐methyladenine (3‐MA), bardoxolone (CDDO), and SRT1460 were obtained from MedChemExpress (MCE) (Monmouth Junction, NJ, USA). MDA, GSH, and SOD assay kits were purchased from Jiancheng Bioengineering Institute (Nanjing). The ROS fluorescence detection kit, mitochondrial ROS detection kit, Hoechst staining kit, mitochondrial red fluorescence probe, ATP assay kit, and JC‐1 MMP detection kit were all obtained from Beyotime (Shanghai).

### 2.2. Animals

This study used 40 healthy male C57BL/6 mice, with an average weight of 18 ± 2 g, obtained from Liaoning Changsheng Biotechnology Co., Ltd. All experimental procedures were rigorously executed in accordance with the National Guidelines for Laboratory Animal Welfare and received formal ethical clearance (Approval No. ys085) from the Institutional Animal Care and Use Committee of the First Affiliated Hospital, Harbin Medical University. All subjects were maintained under controlled environmental conditions with ambient temperature regulated at 21°C–23°C, relative humidity maintained at 50% ± 10%, and standardized photoperiod cycles (12‐h light/dark illumination). Animals were provided ad libitum access to sterile water and standard laboratory chow throughout the experimental period.

### 2.3. Treatments

The dosing for the experimental animals was calculated based on the human dosage [22]. The selection of the ATO dose was based on our preliminary experiments and previous research. The specific groupings were as follows: (a) Control group: Mice received an intraperitoneal injection of an equal volume of saline (0.9%); (b) ATO group: Mice received an intraperitoneal injection of 5 mg/kg of ATO along with 10 mL/kg of saline; (c) EMPA group: Mice were gavaged with 4 mg/kg EMPA; (d) EMPA plus ATO (E + A) group: Mice received an intraperitoneal injection of 5 mg/kg ATO and were also gavaged with 4 mg/kg EMPA.

### 2.4. Cell Culture and Treatments

The HEK293T cells were maintained in Dulbecco’s modified Eagle medium (DMEM) supplemented with 10% fetal bovine serum (FBS) within a humidified incubator at 37°C under 5% CO2 atmosphere. Cellular viability was quantified using the Cell Counting Kit‐8 assay (CCK‐8, Beyotime Biotechnology, Shanghai). HEK293T cells were randomly divided into the following groups: (a) Control group: treated only with the culture medium for 24 h; (b) EMPA group: treated with 4 μm EMPA for 24 h; (c) ATO group: treated with 6 μm ATO for 24 h; and (d) ATO + EMPA group: treated with a combination of 6 μm ATO and 4 μm EMPA for 24 h.

### 2.5. Histopathologic Assay and Measurement of Serum Creatinine (CREA) and Blood Urea Nitrogen (BUN)

Renal tissues were fixed in 4% paraformaldehyde solution (24 h minimum) followed by conventional paraffin embedding. Histological sections were prepared at 4 μM thickness using a microtome and subjected to hematoxylin–eosin (H&E) staining and Masson’s trichrome collagen detection. Serum biochemical parameters including BUN and CREA were quantitatively analyzed using standardized commercial assay kits (Nanjing Jiancheng Bioengineering Institute, China) following the manufacturer’s protocols.

### 2.6. Measurement of Antioxidants and ROS

Oxidative stress biomarkers including ROS, MDA, GSH, and SOD were quantitatively assessed through spectrophotometric analysis using standardized commercial kits (Jiancheng Bioengineering Institute, Nanjing, China). Intracellular ROS levels were determined using a fluorescent probe‐based assay per manufacturer’s guidelines. Post‐treatment cells underwent PBS washing and subsequent incubation with 10 μM DCFH‐DA in complete darkness (37°C, 30 min), followed by fluorescence microscopy imaging for oxidative stress visualization.

### 2.7. Measurement of MMP

The MMP was evaluated utilizing the JC‐1 MMP detection kit along with the MitoTracker Red reagent. Various groups of HEK293T cells were plated in 6‐well plates. Following the aforementioned treatment, cells underwent two washes with PBS and were subsequently incubated in the dark with the JC‐1 working solution at 37°C for a duration of 30 min. After incubation, the cells were washed twice with JC‐1 buffer, and fluorescence images were obtained using a fluorescence microscope. A reduction in the relative fluorescence ratio (red/green fluorescence intensity) signifies a decrease in MMP.

### 2.8. Measurement of Mitochondrial ROS

MitoSOX Red reagent is used to analyze mitochondrial ROS levels. According to the kit instructions, the appropriate working solution is prepared. When the cells are cultured in a cell culture plate or dish to a certain density, the cell culture medium is removed, and the prepared working solution is added. The cells are incubated at 37°C for 15–30 min. After removing the working solution, fresh prewarmed cell culture medium at 37°C is added. Observation is then conducted using a fluorescence microscope.

### 2.9. Western Blot Analysis

Renal tissues and HEK293T cells were homogenized in RIPA buffer supplemented with protease inhibitor cocktail. After measuring the protein concentration using a BCA kit, the protein samples were denatured in 5 × loading buffer. The protein samples were loaded onto a 10% SDS‐PAGE, followed by transfer to a PVDF membrane. The membrane was blocked with 5% nonfat milk at room temperature for 2 h and then incubated overnight at 4°C with the following primary antibodies: Bax (1:1000, abways), Bcl‐2 (1:1000, abways), Caspase‐3 (1:1000, abways), P62 (1:1000, abways), LC3B (1:1000, abmart), SIRT1 (1:1000, abways), Nrf2 (1:2000, ABclonal), NQO1 (1:1000, ABclonal), Akt (1:1000, MCE), p‐Akt (1:1000, MCE), and β‐actin (1:2000, Affinity Biosciences). After incubation with the primary antibodies, the membrane was washed three times with PBST and then incubated with the corresponding secondary antibody (1:2000, Invitrogen) for 1 h. The PVDF membrane was scanned using a gel imaging system, and protein bands were quantified using ImageJ software, with β‐actin serving as an internal reference.

### 2.10. Quantitative Real‐Time PCR (qRT‐PCR)

Total RNA was isolated from HEK293T cells using TRIzol reagent (Invitrogen, CA, USA). Complementary DNA (cDNA) synthesis was performed with PrimeScript RT Master Mix (Toyobo, Osaka, Japan). qRT‐PCR was conducted to analyze SIRT1 mRNA levels using the SYBR Green detection system, with GAPDH as an endogenous control. Amplification reactions were performed in triplicate using the following conditions: 95°C for 30 s, 40 cycles of 95°C for 5 s, and 60°C for 30 s. Relative quantification was calculated using the 2^−ΔΔCT^ method with normalization to GAPDH expression.

### 2.11. Molecular Docking

Molecular docking was performed utilizing AutoDock Vina 1.2.5 and AutoDock 1.5.6 Tools. This process encompassed protein preparation, ligand docking, and subsequent result analysis using PyMOL 3.0.2. Key steps included the assignment of charges, the addition of hydrogen atoms, the definition of rotatable bonds, and the generation of a receptor grid file for ligand docking.

### 2.12. Statistical Analysis

All experimental data are expressed as mean ± standard deviation (SD). Statistical analyses were performed by a two‐tailed unpaired Student’s *t*‐test for two experimental groups, while one‐way analysis of variance (ANOVA) was applied for multigroup comparisons. Statistical significance was defined as ^∗^ *p* < 0.05 throughout all experiments.

## 3. Result

### 3.1. EMPA Alleviates Kidney Injury Induced by ATO in C57 Mice

Body weight in mice exhibited progressive elevation throughout the intervention period, although no significant intergroup differences were observed at any measured time points (Figure 1(a)). Blood glucose concentrations remained comparable across all experimental groups at both baseline and postintervention assessments (Figures 1(b), 1(c)). Histopathological analysis demonstrated that ATO administration induced pronounced renal pathology, including tubular epithelial edema, glomerular capillary congestion, and elevated collagen deposition, all of which were substantially attenuated by EMPA treatment (Figures 1(d), 1(e)). Quantitative evaluation revealed a significant increase in the left renal weight index (*p* < 0.05) in the ATO group compared to the control group (Figure 1(f)). Furthermore, the serum levels of BUN (*p* < 0.05) and CREA in the EMPA + ATO group were reduced compared to the ATO group (Figures 1(g), 1(h)).

FIGURE 1EMPA alleviates kidney injury induced by ATO in C57 mice. (a) Weight changes of mice during the intervention from Days 0–28; (b) fasting blood glucose values of mice before the experiment; (c) fasting blood glucose values of mice after the experiment; (d) representative images of renal tissue stained with HE. Scale bar = 100 μm; (e) representative images of renal tissue stained with Masson. Scale bar = 20 μm; (f) left kidney weight index of mice; (g) effects of ATO and EMPA on serum BUN levels; (h) effects of ATO and EMPA on serum CRE levels. Values are expressed as mean ± standard deviation. ^∗^ *p* < 0.05, ^∗∗^ *p* < 0.01, ^∗∗∗^ *p* < 0.001 compared with the control group; ^#^ *p* < 0.05, ^##^ *p* < 0.01, ^###^ *p* < 0.001 compared with the ATO group.

**Figure:**
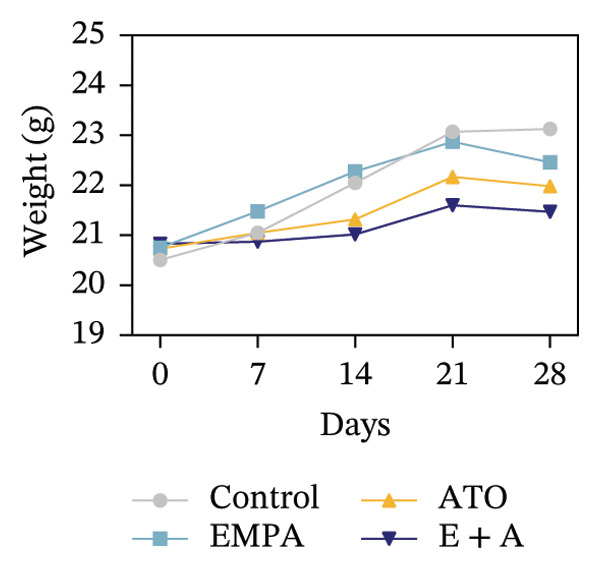
(a)

**Figure:**
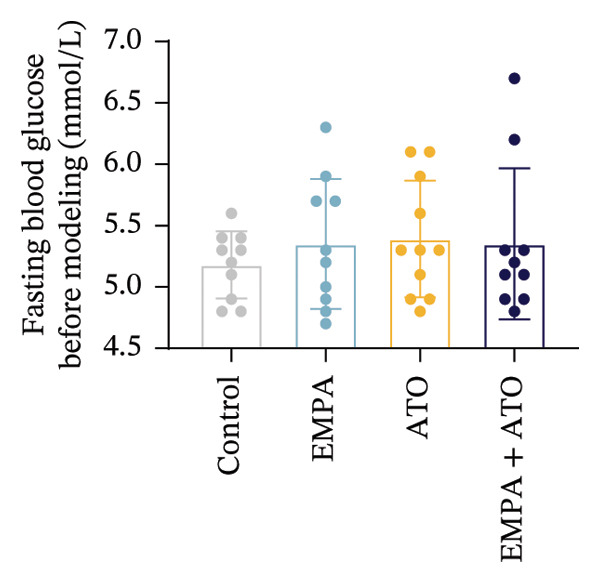
(b)

**Figure:**
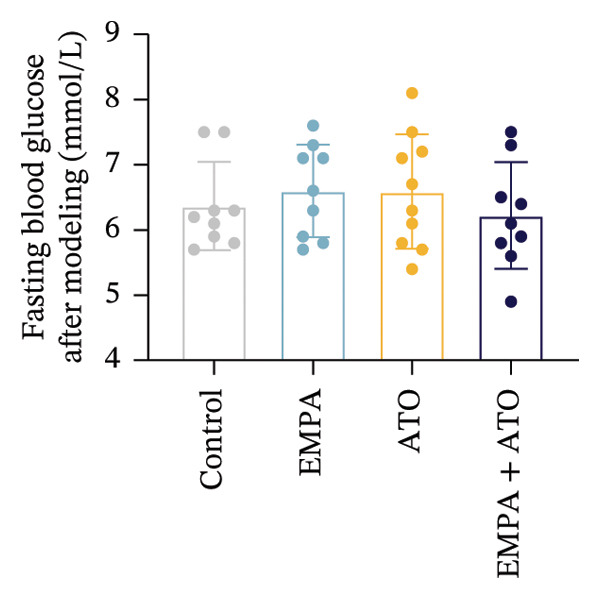
(c)

**Figure:**
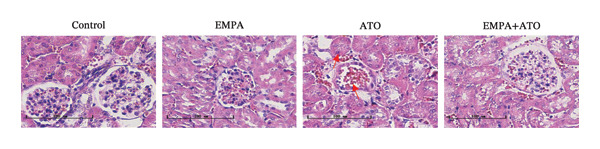
(d)

**Figure:**
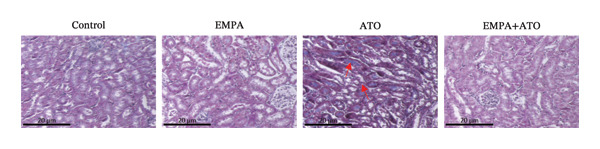
(e)

**Figure:**
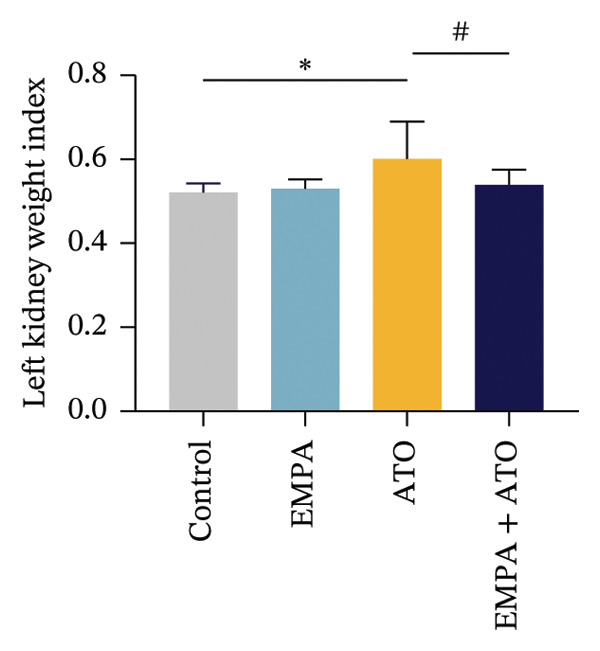
(f)

**Figure:**
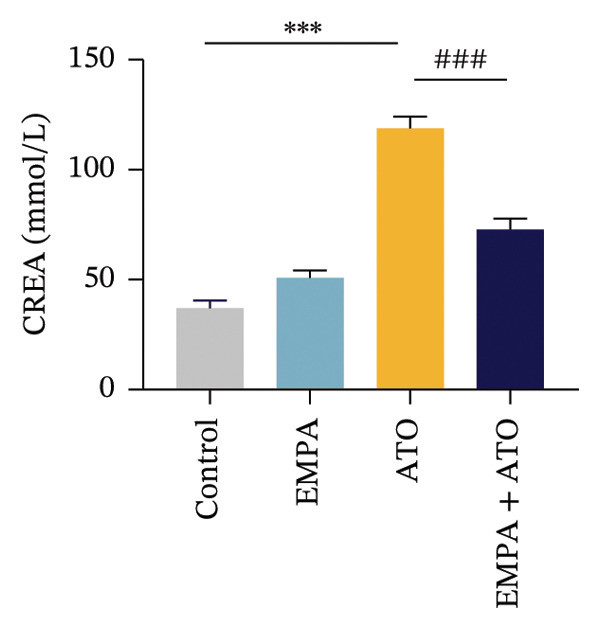
(g)

**Figure:**
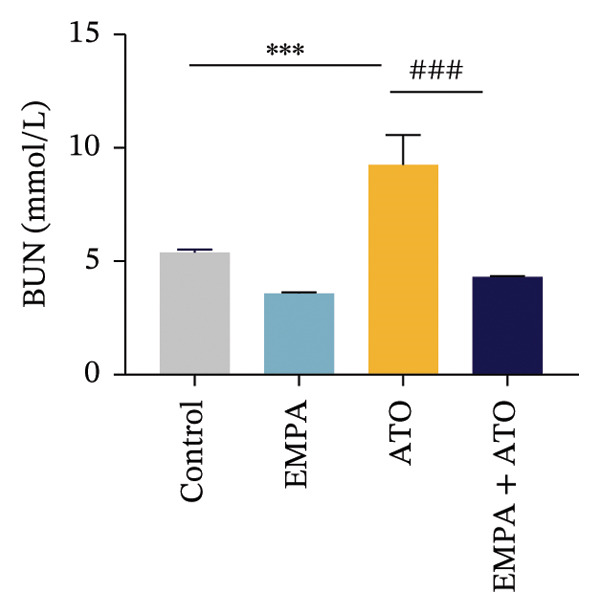
(h)

### 3.2. EMPA Alleviated Oxidative Stress and Apoptosis Induced by ATO in the Kidney of C57 Mice

Comparative analysis revealed pronounced oxidative stress induction in ATO‐treated mice, evidenced by significantly elevated ROS accumulation in renal tissues (Figures 2(a), 2(b)) and increased MDA levels (*p* < 0.05) compared to controls. EMPA intervention effectively mitigated these oxidative perturbations, demonstrating significant reductions in both ROS (*p* < 0.05) and MDA concentrations (Figures 2(b), 2(c)). Concurrently, EMPA administration enhanced systemic antioxidant capacity through marked elevation of SOD (*p* < 0.05) and GSH activity (Figures 2(d), 2(e)).

FIGURE 2EMPA alleviated oxidative stress and apoptosis induced by ATO in the kidney of C57 mice. (a‐b) Mice kidney tissue through DCFH‐DA dyeing evaluation level of ROS. Scale bar = 100 μm. (c) The ATO and EMPA effects on the serum GSH level of mice. (d) The ATO and EMPA effects on the serum MDA levels of mice. (e) Effects of ATO and EMPA on serum SOD levels (f). Western blot analysis of the effects of EMPA and ATO on the expression of Caspase‐3, Bcl‐2, and Bax (*n* = 3). (g–i) Caspase‐3, Bax, and Bcl‐2 in kidneys of mice tissue expression changes. ^∗^ *p* < 0.05, ^∗∗^ *p* < 0.01, ^∗∗∗^ *p* < 0.001 compared with the control group; ^#^ *p* < 0.05, ^##^ *p* < 0.01, ^###^ *p* < 0.001 compared with the ATO group.

**Figure:**
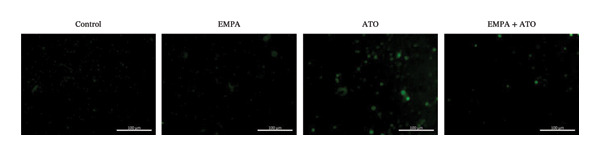
(a)

**Figure:**
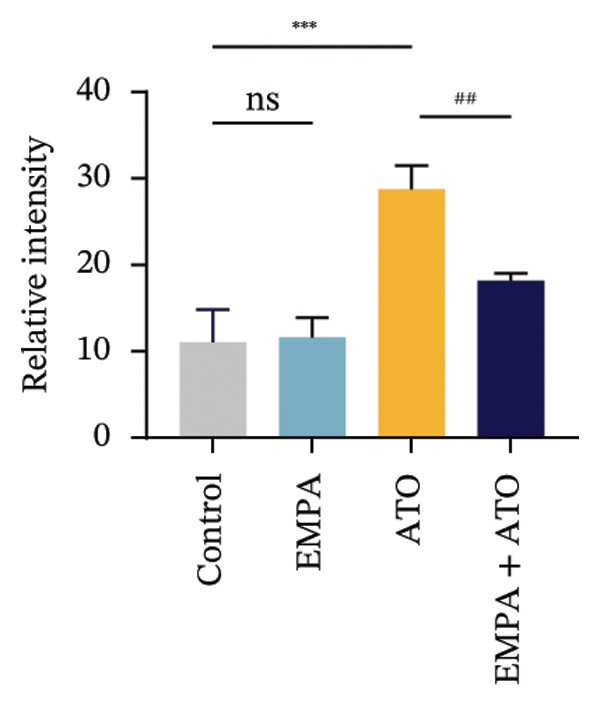
(b)

**Figure:**
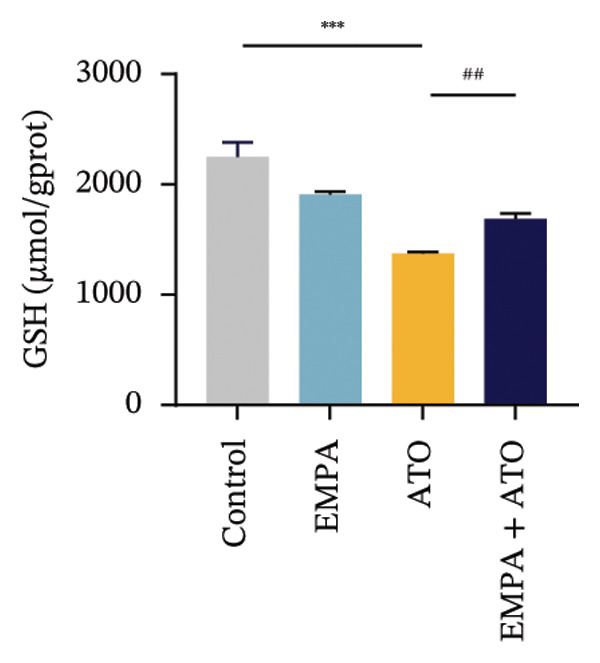
(c)

**Figure:**
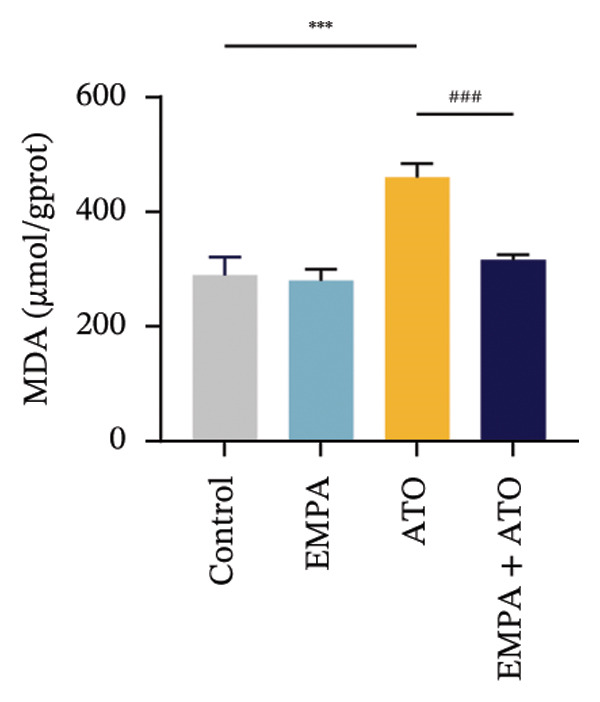
(d)

**Figure:**
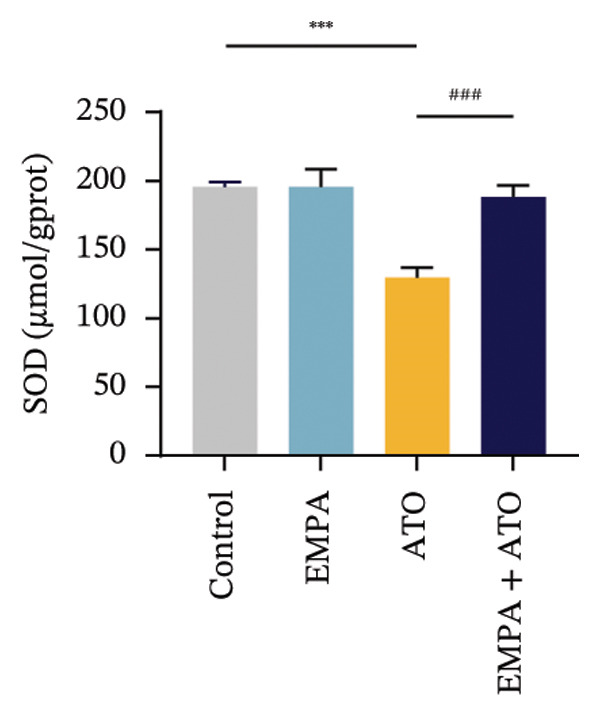
(e)

**Figure:**
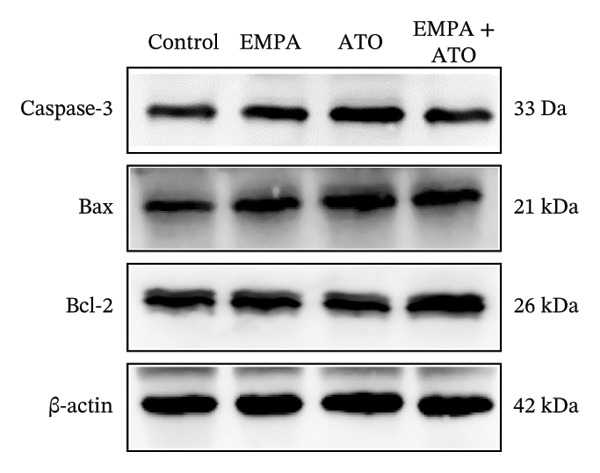
(f)

**Figure:**
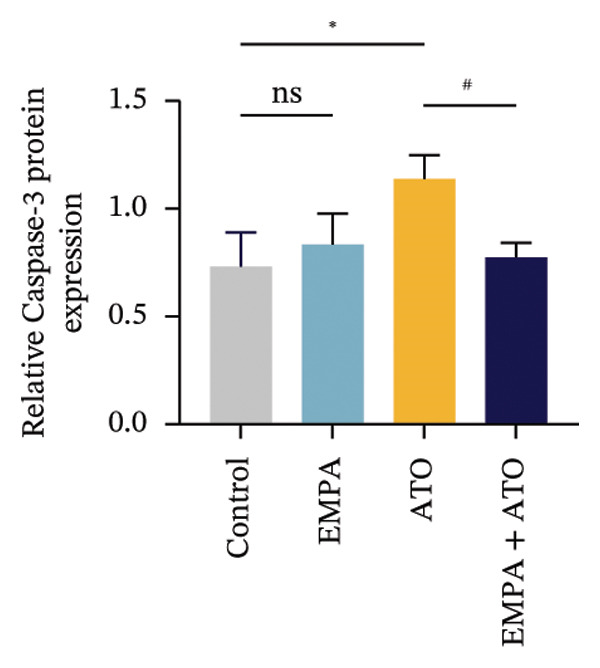
(g)

**Figure:**
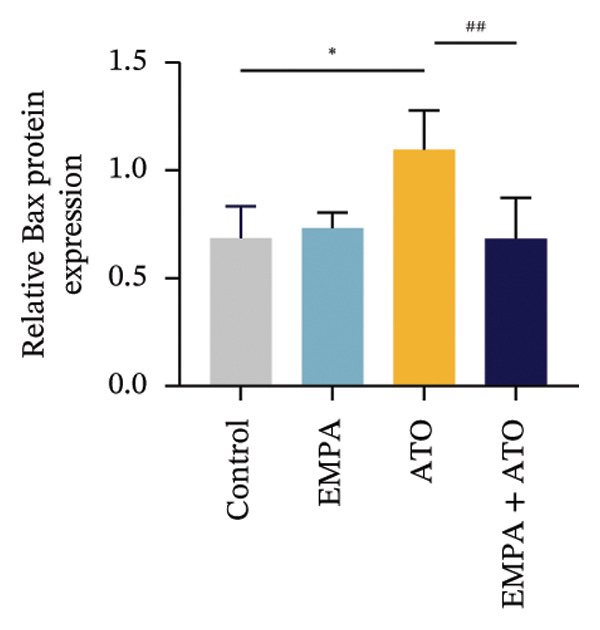
(h)

**Figure:**
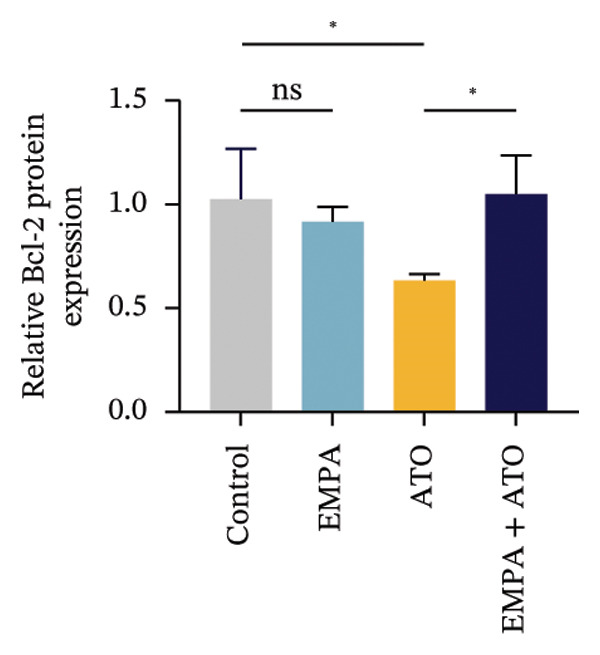
(i)

Oxidative stress may ultimately lead to cell apoptosis. To investigate the downstream consequences of oxidative damage, we evaluated apoptosis‐related protein expression via Western blot analysis (Figure 2(f)). Renal tissues from ATO‐exposed mice exhibited significantly upregulated proapoptotic mediators (Bax and Caspase‐3) coupled with suppressed Bcl‐2 expression relative to controls (Figures 2(g), 2(h), 2(i)). EMPA administration substantially reversed these apoptotic alterations, normalizing Bax and Caspase‐3 expression while restoring Bcl‐2 levels (*p* < 0.05).

### 3.3. EMPA Activates the SIRT1/Akt/Nrf2 Signaling Pathway and Inhibits Autophagy in the Kidney of C57 Mice

Mechanistic investigations into SIRT1 pathway modulation demonstrated that EMPA significantly enhanced SIRT1 protein expression (*p* < 0.05) while concurrently suppressing Akt phosphorylation and reducing Nrf2/NQO1 axis activation (Figures 3(a), 3(b)). Given the pathophysiological interconnection between autophagy and apoptosis, subsequent evaluation of autophagic markers revealed ATO‐induced dysregulation characterized by elevated LC3B‐II accumulation and diminished p62 expression (Figures 3(c), 3(d)). EMPA co‐treatment effectively normalized these autophagic perturbations (*p* < 0.05), suggesting therapeutic modulation of autophagy homeostasis.

FIGURE 3EMPA activates the SIRT1/Akt/Nrf2 signaling pathway and inhibits autophagy in the kidney of C57 mice. (a) Changes in the expression of SIRT1, P‐Akt, and Akt in mice kidney tissue. (b) Relative expression levels of SIRT1, P‐Akt, and Akt. (c) Western blotting was used to detect changes in the expression of Nrf2 and NQO1 in renal tissue. (d) Relative expression levels of Nrf2 and NQO1. (e) Western blotting was used to detect changes in the expression of P62 and LC3B‐II proteins in renal tissue. (f) Relative expression levels of P62 and LC3B‐II. Values are expressed as mean ± standard deviation. ^∗^ *p* < 0.05, ^∗∗^ *p* < 0.01, ^∗∗∗^ *p* < 0.001 compared with the control group; ^#^ *p* < 0.05, ^##^ *p* < 0.01, ^###^ *p* < 0.001 compared with the ATO group.

**Figure:**
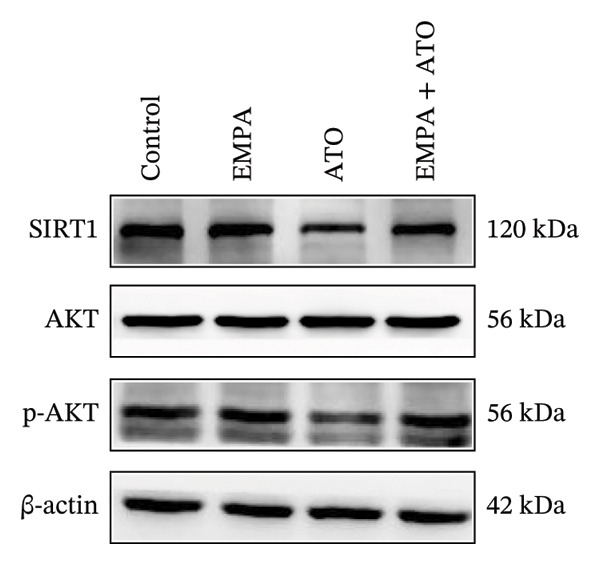
(a)

**Figure:**
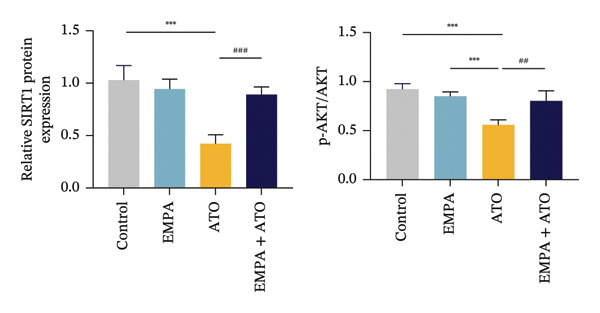
(b)

**Figure:**
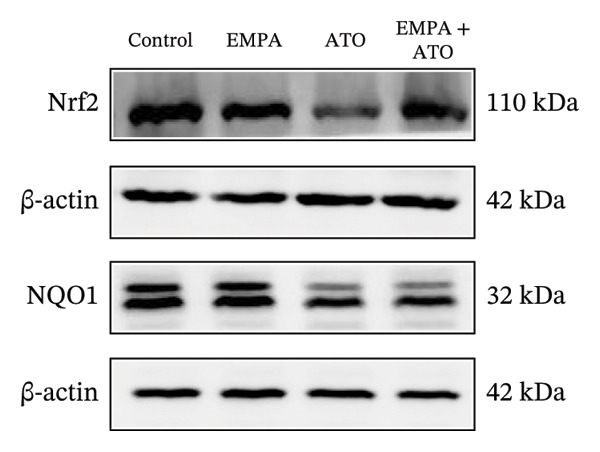
(c)

**Figure:**
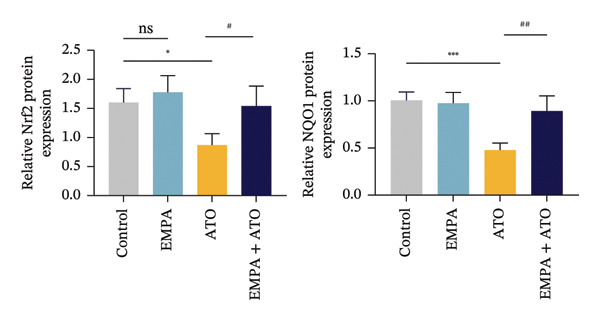
(d)

**Figure:**
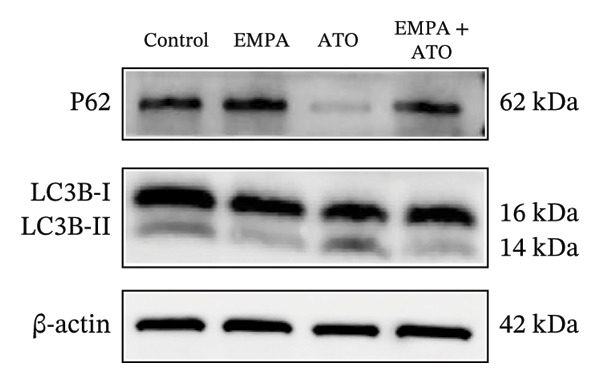
(e)

**Figure:**
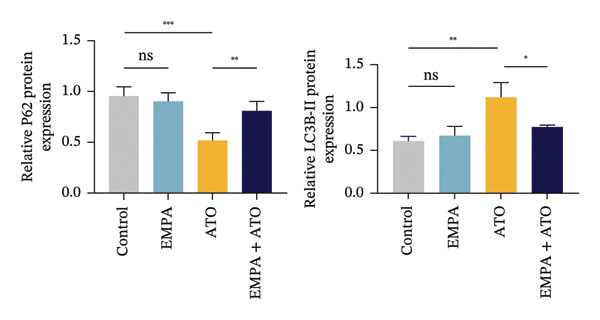
(f)

### 3.4. EMPA Improves the Decrease in Cell Viability and Morphological Changes Induced by ATO In Vitro

HEK293T cells were treated with different concentrations of ATO (2–20 μm) for 24 h, and cell viability was assessed using the CCK8 assay. The results showed that ATO reduced cell viability in a dose‐dependent manner. In this experiment, we determined the ATO treatment concentration to be 6 μm (Figure 4(a)). Subsequently, different concentrations of EMPA were combined with 6 µM ATO and incubated for 24 h. It was found that EMPA exhibited the most significant protective effect at a concentration of 4 μm, so this was chosen as the working concentration for EMPA (Figure 4(b)). A low dose of the autophagy inhibitor 3‐MA can alleviate the decrease in cell viability induced by ATO (Figure 4(c)). The morphological changes of HEK293 cells under the influence of ATO and EMPA were observed using a bright‐field inverted microscope. HEK293 cells showed normal morphology when not exposed to ATO or treated only with EMPA. Exposure to ATO resulted in significant morphological changes. After combined treatment with EMPA, the cell morphology improved significantly (Figure 4(d)). Hoechst staining results showed that the nuclei of cells treated with ATO exhibited dense and concentrated staining compared to the control group. Treatment with EMPA significantly reduced the nuclear condensation induced by ATO.

FIGURE 4EMPA improves the decline in cell viability and morphological changes caused by ATO in vitro. (a) ATO exhibits a dose‐dependent inhibitory effect on the viability of HEK293T cells (*n* = 3). (b) EMPA significantly upregulated the vitality of HEK293T cells in a dose‐dependent manner (*n* = 3). (c) Low‐dose autophagy inhibitor 3‐MA can improve the decrease in cell viability induced by ATO. (d) Light‐field inverted microscopy was used to observe morphological changes in HEK293T cells induced by arsenic exposure. Scale bar = 50 μm. (e) Hoechst staining results (*n* = 3). Scale bar = 100 μm. Values are expressed as mean ± standard deviation. ^∗^ *p* < 0.05, ^∗∗^ *p* < 0.01, ^∗∗∗^ *p* < 0.001 compared with the control group; ^#^ *p* < 0.05, ^##^ *p* < 0.01, ^###^ *p* < 0.001 compared with the ATO group.

**Figure:**
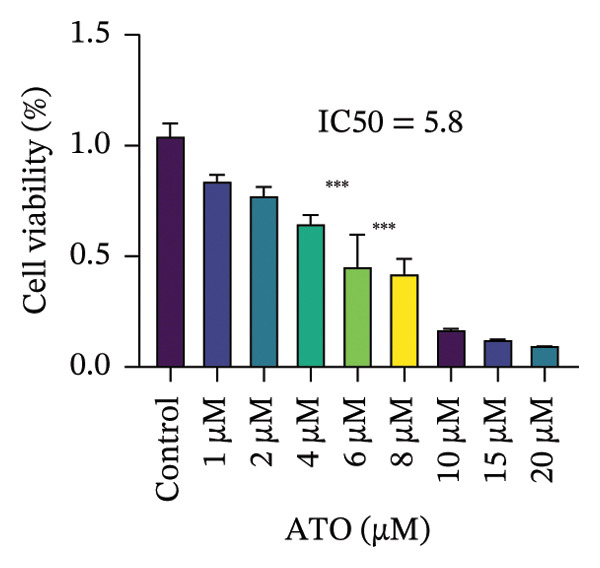
(a)

**Figure:**
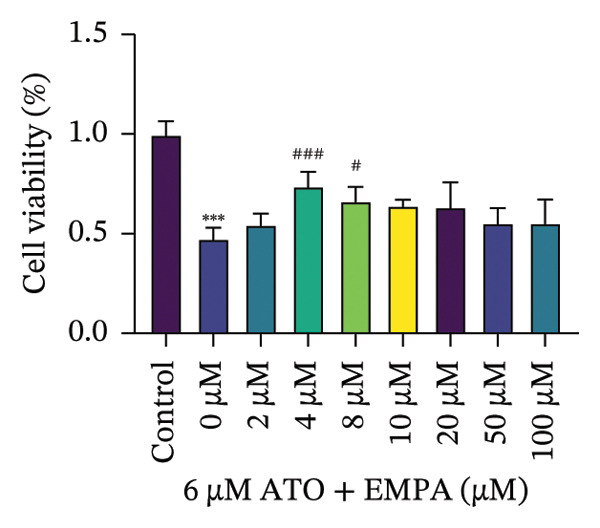
(b)

**Figure:**
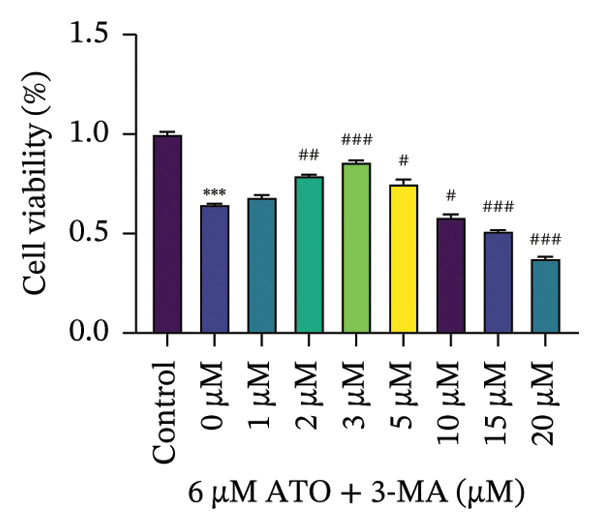
(c)

**Figure:**
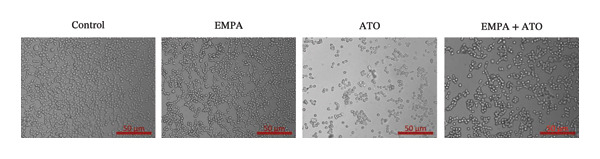
(d)

**Figure:**
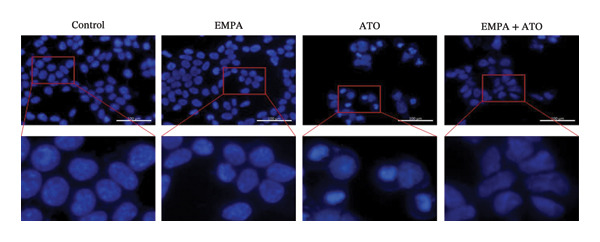
(e)

### 3.5. EMPA Alleviates Oxidative Stress and Cell Apoptosis Induced by ATO In Vitro

Evaluate the accumulation of ROS in HEK293T cells among different treatment groups. The intensity of green fluorescence images can represent the level of ROS (Figure 5(a)). Compared to the control group, ROS levels were significantly increased in ATO‐treated HEK293T cells, while combined treatment with EMPA significantly reduced ROS levels (Figure 5(b)). The MDA levels in the ATO group were significantly higher than in the control group, while GSH and SOD activity were significantly lower. Combined treatment with EMPA significantly reduced MDA levels while increasing GSH and SOD activity (Figures 5(c), 5(d), 5(e)) (*p* < 0.05). In summary, these results indicate that EMPA can improve ATO‐induced oxidative stress in HEK293T cells. The Western blot results show that ATO reduces Bcl‐2 expression while increasing Caspase‐3 and Bax protein levels. These results suggest that ATO has a significant proapoptotic effect. Compared to the ATO group, Bcl‐2 levels increased and Caspase‐3 and Bax levels decreased (*p* < 0.05) after combined EMPA treatment (Figures 5(g), 5(h), 5(i)), indicating that EMPA alleviates ATO‐induced cell apoptosis.

FIGURE 5EMPA alleviates oxidative stress and cell apoptosis induced by ATO in vitro. (a) Results of DCFH‐DA staining for HEK293T cells. (b) Fluorescence intensity of DCFH‐DA staining in HEK293T cells. Scale bar = 100 μm. (c) Impact of ATO and EMPA on intracellular GSH levels. (d) Impact of ATO and EMPA on intracellular SOD levels. (e) Impact of ATO and EMPA on intracellular MDA levels. (f) Western blot analysis assessing the influence of EMPA on the expression levels of Caspase‐3, Bcl‐2, and Bax (*n* = 3). (g–i) Changes in the expression of Caspase‐3, Bax, and Bcl‐2 in HEK293T cells. Values are expressed as mean ± standard deviation. ^∗^ *p* < 0.05, ^∗∗^ *p* < 0.01, ^∗∗∗^ *p* < 0.001 compared with the control group; ^#^ *p* < 0.05, ^##^ *p* < 0.01, ^###^ *p* < 0.001 compared with the ATO group.

**Figure:**
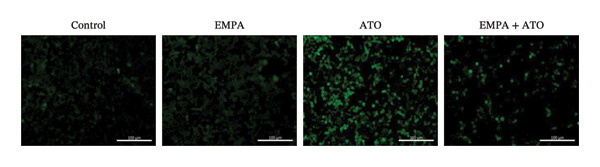
(a)

**Figure:**
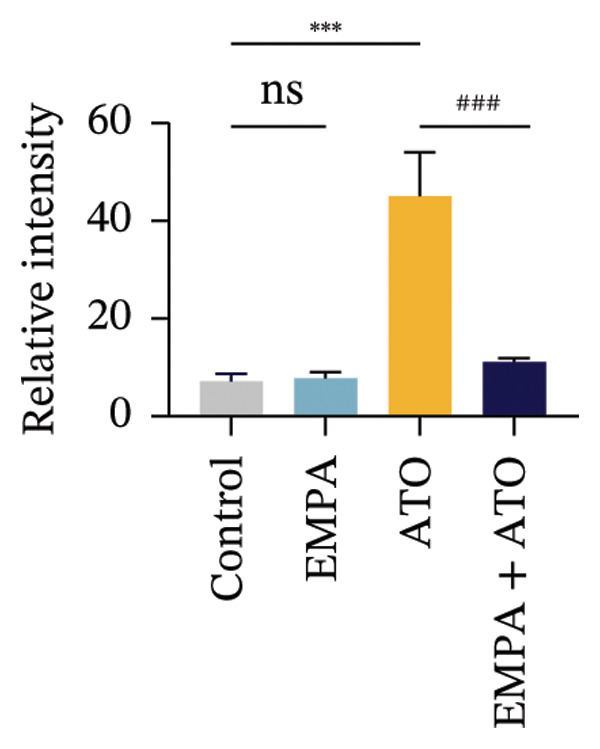
(b)

**Figure:**
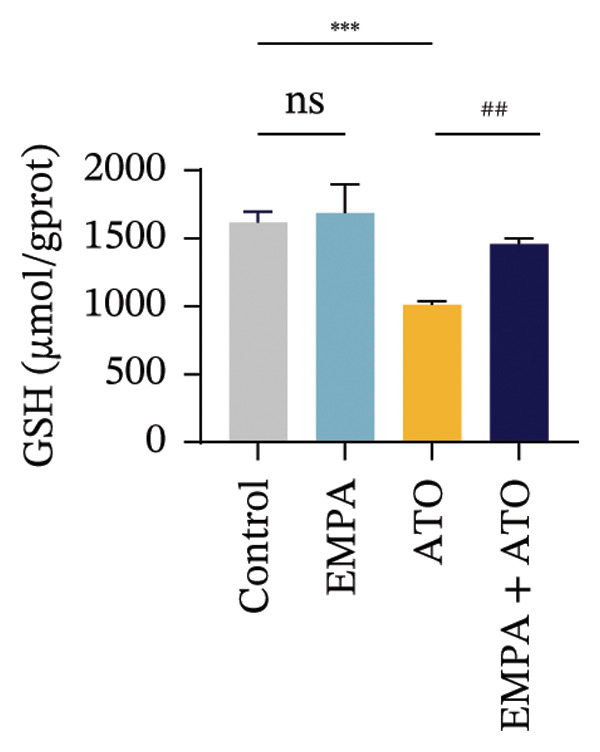
(c)

**Figure:**
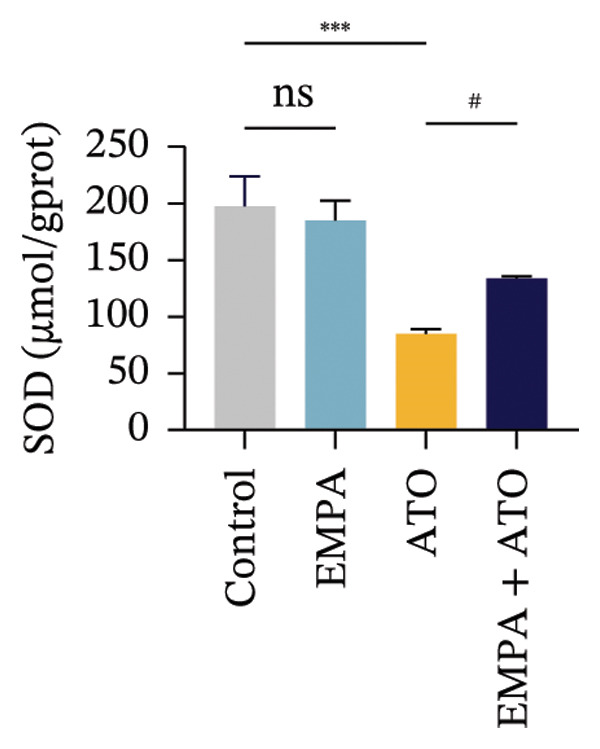
(d)

**Figure:**
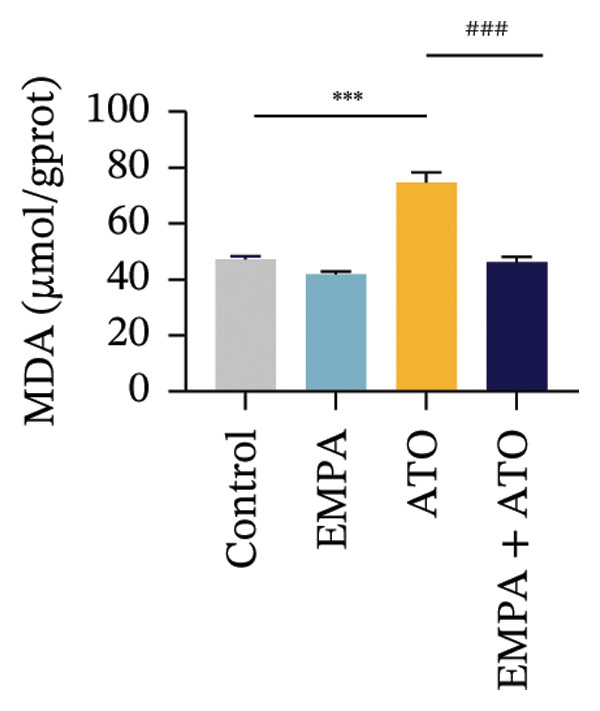
(e)

**Figure:**
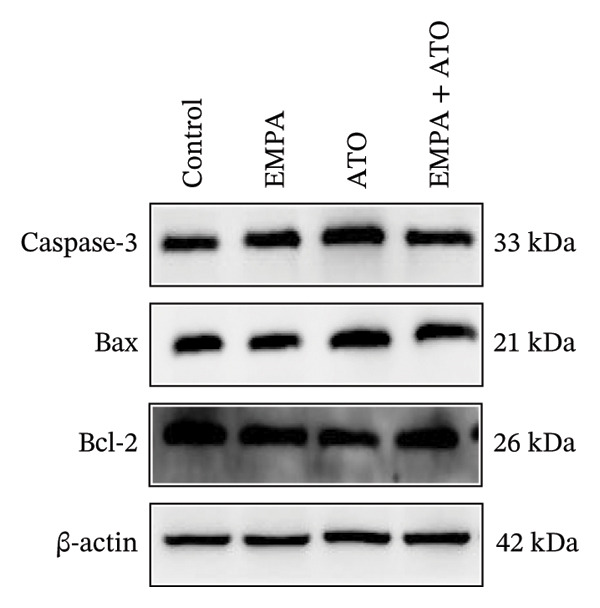
(f)

**Figure:**
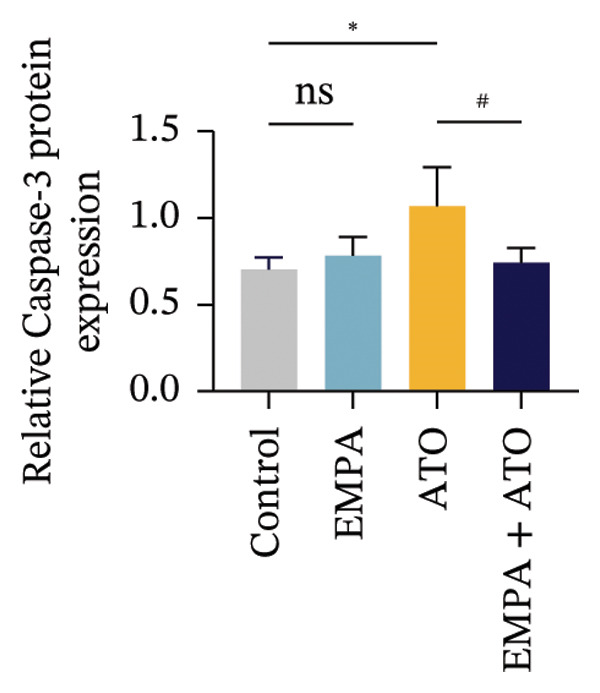
(g)

**Figure:**
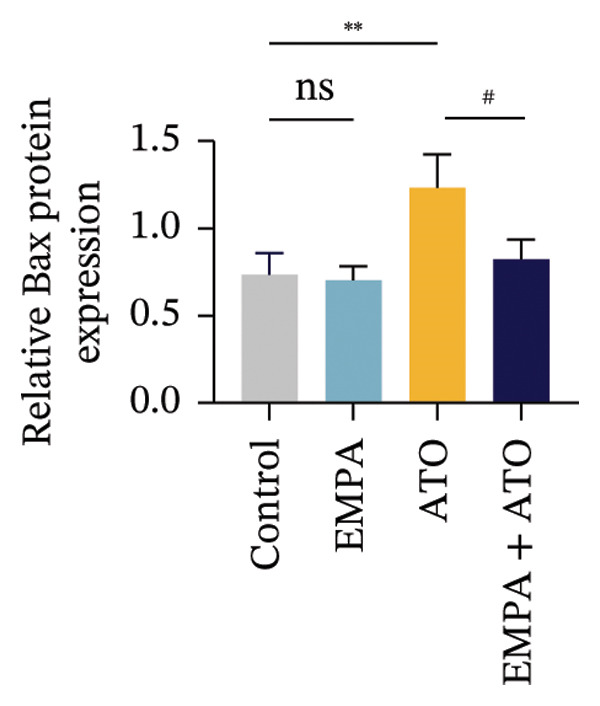
(h)

**Figure:**
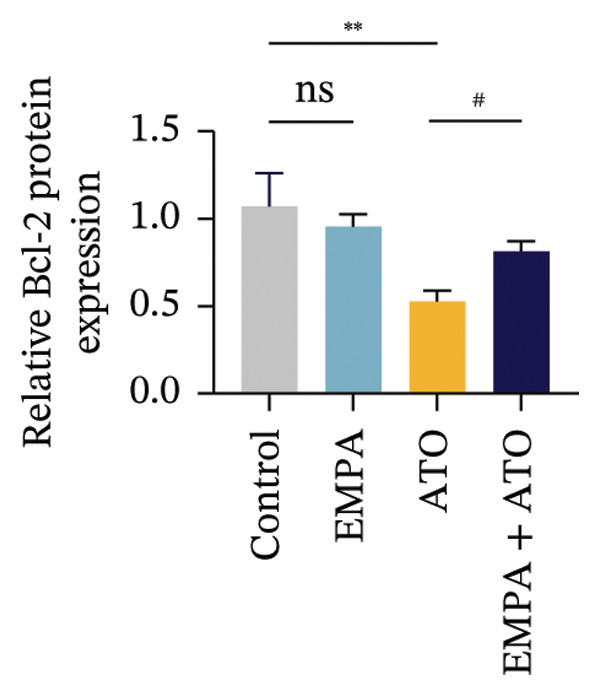
(i)

### 3.6. EMPA Reduces Mitochondrial Superoxide and Preserves Integrity Stimulated by ATO In Vitro

Mitochondrial functional assessments employing JC‐1 staining revealed distinct MMP across treatment groups. In normal control cells, red JC‐1 aggregates are present, indicating hyperpolarized MMP. EMPA has no significant effect on the MMP of normal cells. In the ATO group, green JC‐1 monomers are observed, indicating a loss of MMP. In the combined treatment with EMPA treatment group, MMP is restored to normal levels (Figures 6(a), 6(b)) (*p* < 0.05). MitoTracker staining showed that ATO caused changes in mitochondrial morphology and a decrease in MMP, while EMPA partially alleviated this trend (Figures 6(c), 6(d)) (*p* < 0.05). MitoSOX staining shows that ATO stimulation increases the production of mitochondrial‐dependent ROS, while EMPA treatment reduces the amount of mitochondrial superoxide anions induced by ATO (Figures 6(e), 6(f)). Additionally, EMPA alleviated the decline in ATP production induced by ATO (Figure 6(g)). These findings suggest that EMPA treatment significantly suppresses the formation of mitochondrial superoxide in HEK293T cells stimulated by ATO and protects mitochondrial integrity.

FIGURE 6EMPA reduces mitochondrial superoxide and preserves integrity stimulated by ATO in vitro. (a) Assessment of EMPA’s effect on ATO‐induced dissipation of mitochondrial membrane potential in HEK293T cells loaded with JC‐1, measured using fluorescence microscopy. Scale bar = 20 μm. (b) Quantitative analysis of JC‐1 relative fluorescence (red/green ratio). (c) Evaluation of mitochondrial morphological changes using MitoTracker. Scale bar = 100 μm. (d) Quantification of MitoTracker results. Scale bar = 100 μm. (e) Observation of mitochondrial reactive oxygen species generation using MitoSOX. Scale bar = 100 μm. (f) Quantification of MitoSOX results. (g) ATP relative content measurement. Values are expressed as mean ± standard deviation. ^∗^ *p* < 0.05, ^∗∗^ *p* < 0.01, ^∗∗∗^ *p* < 0.001 compared with the control group; ^#^ *p* < 0.05, ^##^ *p* < 0.01, ^###^ *p* < 0.001 compared with the ATO group.

**Figure:**
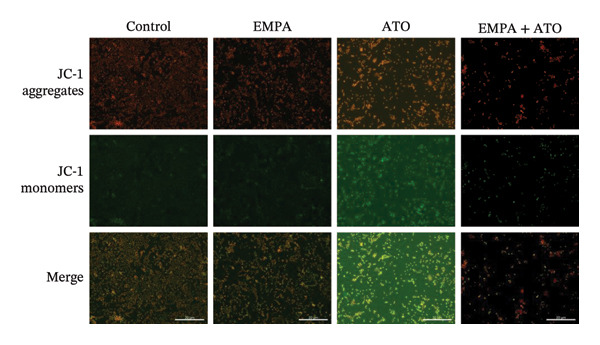
(a)

**Figure:**
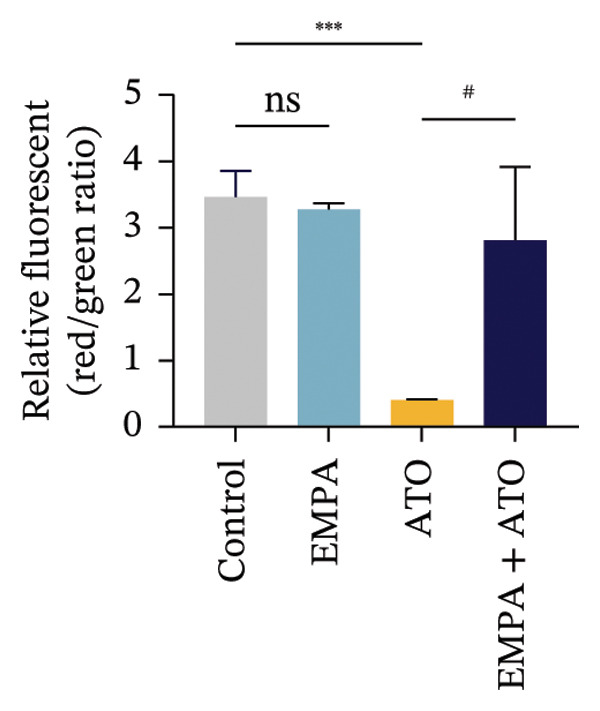
(b)

**Figure:**
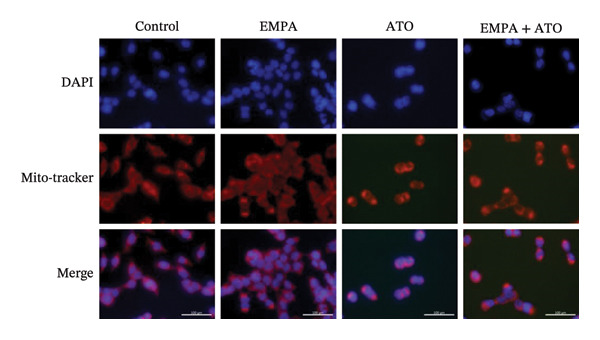
(c)

**Figure:**
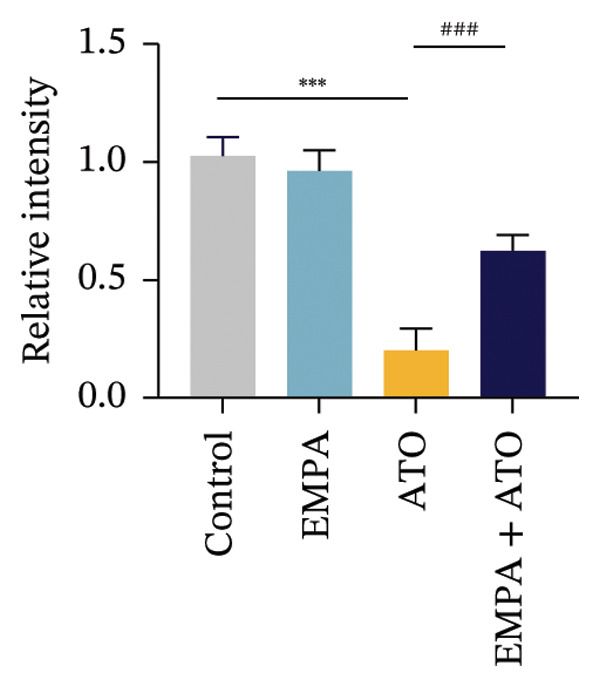
(d)

**Figure:**
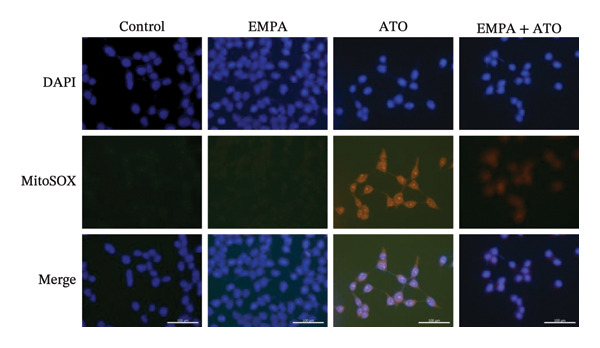
(e)

**Figure:**
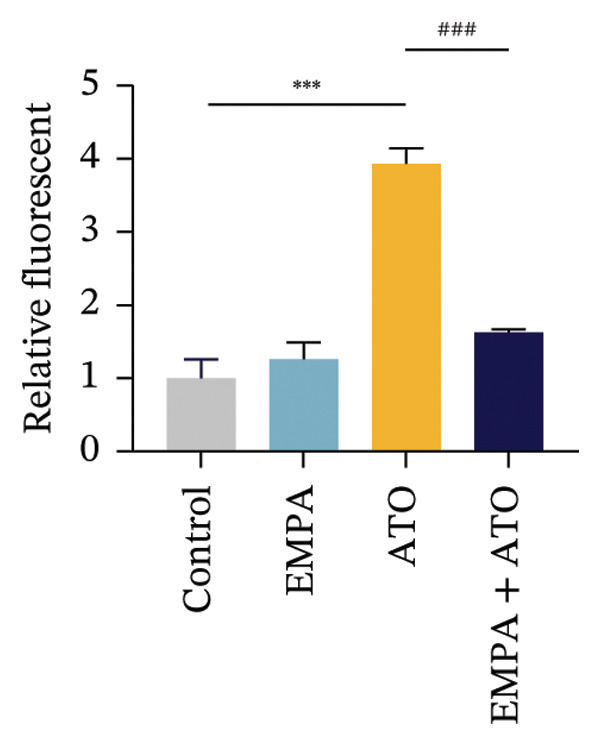
(f)

**Figure:**
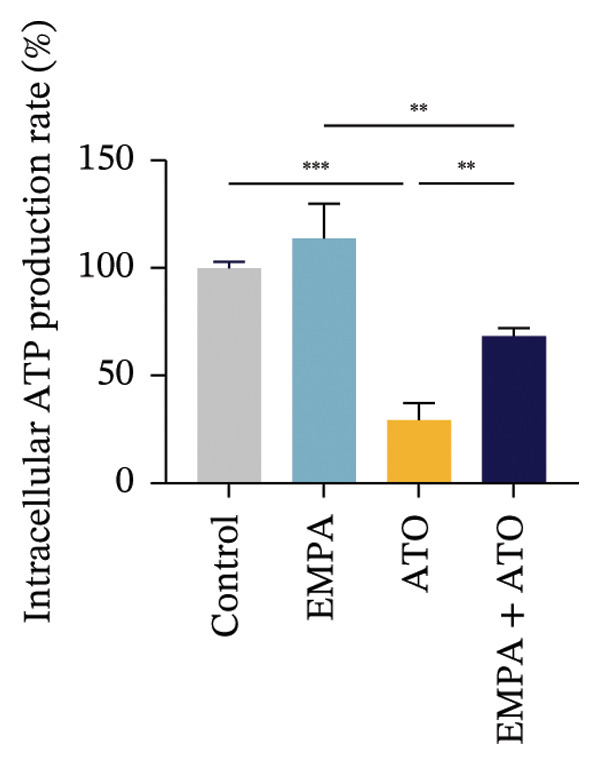
(g)

### 3.7. EMPA Activates the SIRT1/Akt/Nrf2 Signaling Pathway and Inhibits Autophagy In Vitro

The Western blot results demonstrated that EMPA significantly upregulated the expression (*p* < 0.05) of SIRT1, p‐Akt/Akt, and Nrf2, as well as the downstream antioxidant target NQO1 (Figures 7(a), 7(b)). The Western blot results showed that the expression of LC3B‐II was elevated and the expression of P62 protein was decreased in the ATO group cells. In the EMPA + ATO group, the expression of LC3B‐II was downregulated and the expression of P62 protein significantly increased (*p* < 0.05). These results indicate that EMPA can relieve the excessive activation of autophagic flux induced by ATO in HEK293T cells (Figures 7(c), 7(d)). SIRT1 promotes autophagy by mediating post‐translational modifications of autophagy‐initiating proteins and deacetylation of transcription factors [23]. However, this study reveals that ATO suppresses SIRT1 protein expression while enhancing autophagic activity in both murine renal tissues and HEK293T cells. Notably, ATO exhibits no significant impact on SIRT1 mRNA levels in HEK293T cells (Figure 7(g)). Building upon recent findings demonstrating SIRT1 degradation via hyperactivated autophagic pathways during aging [24], our investigation employing Lys05 (an autophagosome–lysosome inhibitor) demonstrates that combination therapy significantly reduces SIRT1 protein degradation compared to ATO monotherapy (Figure 7(h)). These results suggest ATO may accelerate SIRT1 protein degradation through activation of the autophagosome–lysosome pathway.

FIGURE 7EMPA activates the SIRT1/Akt/Nrf2 signaling pathway and inhibits autophagy in vitro. (a) Changes in the expression of SIRT1, P‐Akt, and Akt in HEK293T cells. (b) Relative expression levels of SIRT1, P‐Akt, and Akt. (c) Western blot analysis of changes in the expression of Nrf2 and NQO1 in HEK293T cells. (d) Relative expression levels of Nrf2 and NQO1. (e) Western blot analysis of changes in the expression of P62 and LCB‐II proteins in HEK293T cells. (f) Relative expression levels of P62 and LCB‐II. Values are expressed as mean ± standard deviation. (g) PCR was used to detect changes in the expression of SIRT1 mRNA in HEK293T cells. (h) Western blot analysis of changes in the expression of SIRT1 proteins in HEK293T cells and relative expression levels of SIRT1. ^∗^ *p* < 0.05, ^∗∗^ *p* < 0.01, ^∗∗∗^ *p* < 0.001 compared with the control group; ^#^ *p* < 0.05, ^##^ *p* < 0.01, ^###^ *p* < 0.001 compared with the ATO group.

**Figure:**
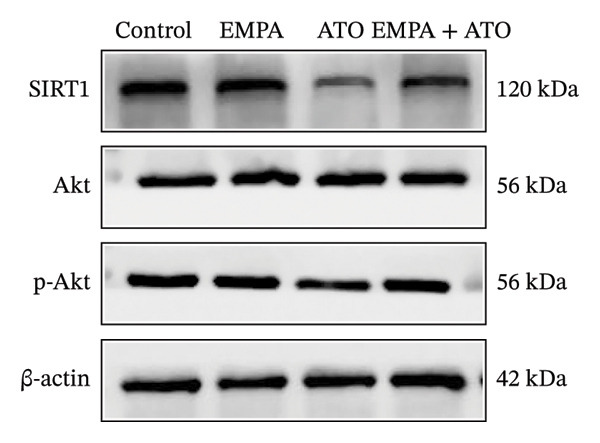
(a)

**Figure:**
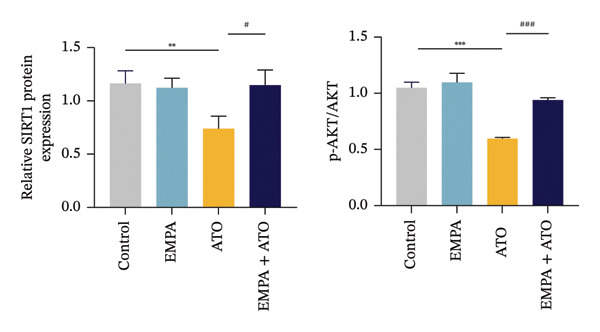
(b)

**Figure:**
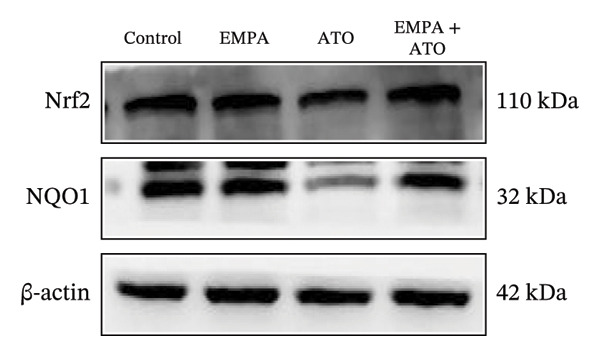
(c)

**Figure:**
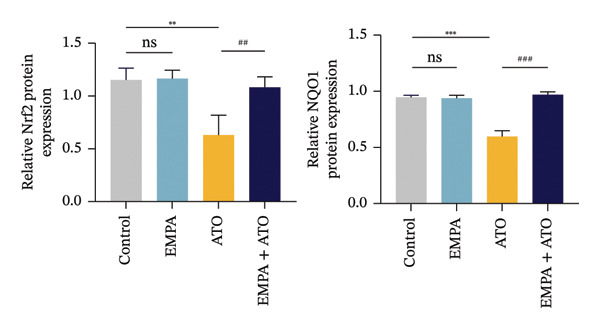
(d)

**Figure:**
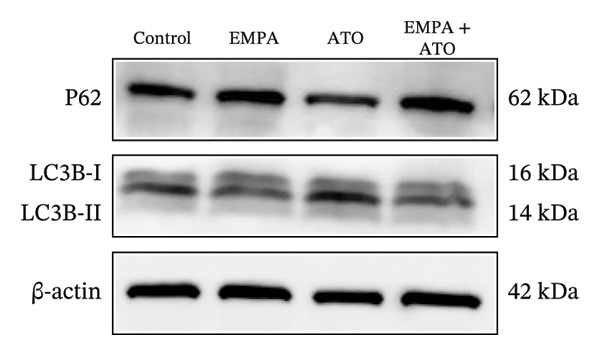
(e)

**Figure:**
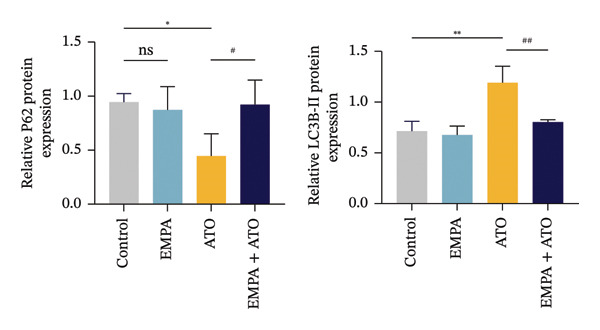
(f)

**Figure:**
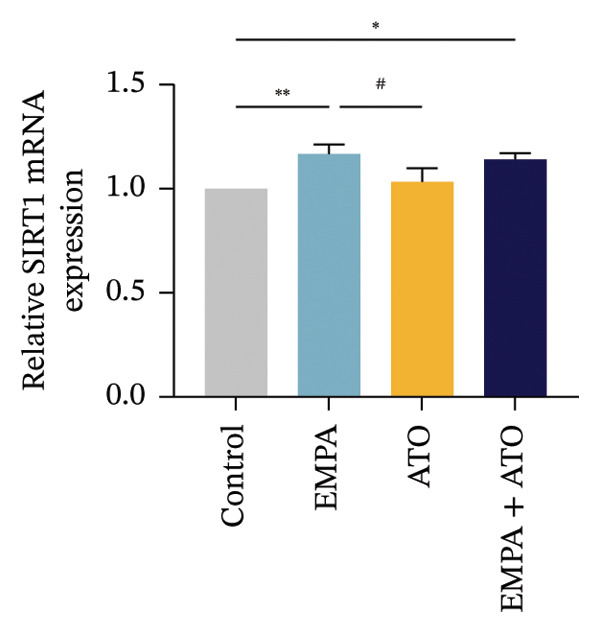
(g)

**Figure:**
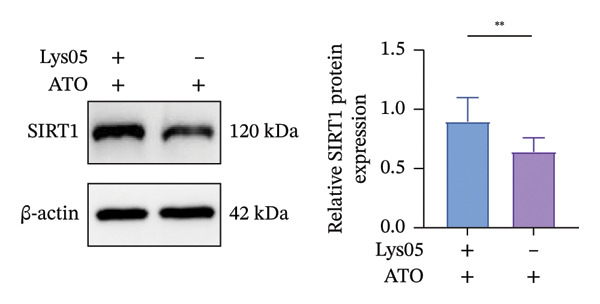
(h)

### 3.8. SIRT1/Akt/Nrf2 Activation Attenuates ATO‐Induced Mitochondrial Oxidative Stress in In Vitro

Sirtinol, a SIRT1 inhibitor, reduces the viability of HEK293T cells (Figure 8(a)). EMPA treatment alleviates the toxic effects of sirtinol (Figure 8(b)). Using molecular docking to simulate the binding of EMPA and sirtinol to SIRT1, molecular docking revealed that EMPA binds to SIRT1 with a binding energy of −9.4 kcal/mol, forming hydrogen bonds with GLN‐345, ALA‐262, and ARG‐274 at distances of 2.6 Å, 2.1 Å, and 2.6 Å, respectively (Figure 8(c)). Sirtinol binds to SIRT1 with a binding energy of −11.4 kcal/mol, forming hydrogen bonds with HIS‐363 at distances of 2.8 Å (Figure 8(d)). These results demonstrate stable and high‐affinity binding between EMPA and SIRT1.

FIGURE 8Molecular docking and experimental validation indicate that SIRT1 is a key target for the therapeutic effects of EMPA. (a) Sirtinol exhibits a dose‐dependent inhibitory effect on the viability of HEK293T cells. (*n* = 3). (b) EMPA can improve the decrease in cell viability induced by sirtinol. (c) Molecular models of EMPA binding to SIRT1. (d) Molecular models of Sirtinol binding to SIRT1.

**Figure:**
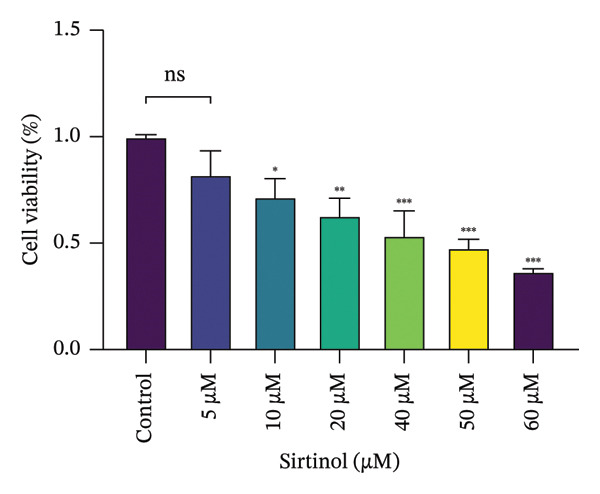
(a)

**Figure:**
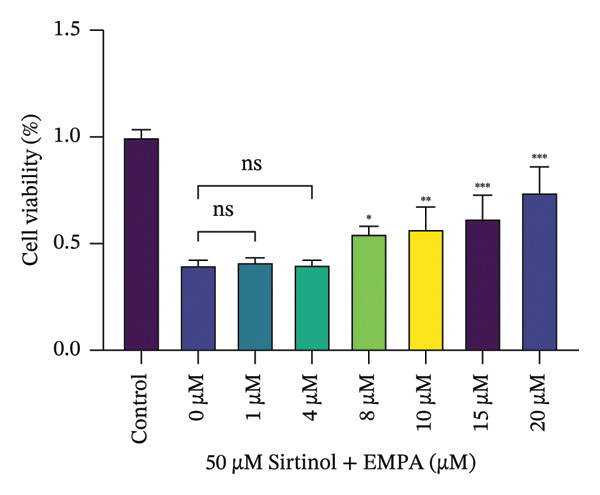
(b)

**Figure:**
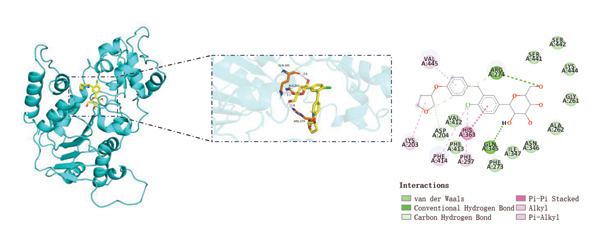
(c)

**Figure:**
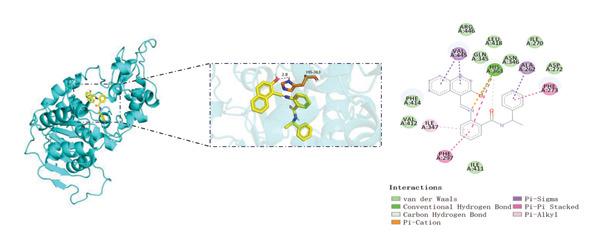
(d)

To further confirm whether the activation of the SIRT1/Akt/Nrf2 pathway protects against ATO‐induced oxidative stress and mitochondrial damage, we treated HEK239T cells with a combination of SIRT1 agonist and Nrf2 agonist along with ATO. SRT1460 is a potent SIRT1 agonist, and bardoxolone (CDDO) is a novel Nrf2 activator. MitoTracker staining showed that compared to the ATO group, mitochondrial morphological changes were significantly alleviated and increased MMP (*p* < 0.05) after treatment with either 3 μM SRT1460 or 1 μM CDDO determined by the dose of these two stimulants according to the instructions (Figure 9(a)). MitoSOX staining indicated that the generation of mitochondrial ROS was significantly reduced (Figure 9(b)) (*p* < 0.05). Western blot analysis detected changes in the SIRT1/Akt/Nrf2 pathway after treatment with SRT1460 and CDDO. The results showed that compared to the ATO group, treatment with either SRT1460 or CDDO resulted in upregulation of SIRT1 expression, increased phosphorylation levels of Akt, upregulation of Nrf2 expression, and upregulation of the Nrf2 downstream protein NQO1 (Figures 9(c), 9(d)) (*p* < 0.05). These results suggest the generation of mitochondrial ROS is partially mitigated and mitochondrial function is protected through the SIRT1/Akt/Nrf2 pathway.

FIGURE 9SIRT1/Akt/Nrf2 activation attenuates ATO‐induced mitochondrial oxidative stress in in vitro. (a) Assessment of mitochondrial morphological changes and quantification using MitoTracker. Scale bar = 100 μm. (b) Observation of mitochondrial reactive oxygen species generation using MitoSOX. Scale bar = 100 μm. (c) Relative expression levels of Nrf2 and NQO1 and their quantification. (d) Relative expression levels and quantification of SIRT1, P‐Akt, and Akt. Values are expressed as mean ± standard deviation. ^∗^ *p* < 0.05, ^∗∗^ *p* < 0.01, ^∗∗∗^ *p* < 0.001 compared with the control group; ^#^ *p* < 0.05, ^##^ *p* < 0.01, ^###^ *p* < 0.001 compared with the ATO group.

**Figure:**
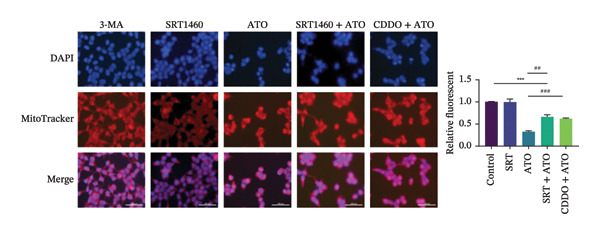
(a)

**Figure:**
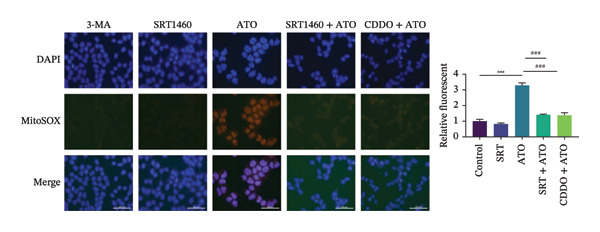
(b)

**Figure:**
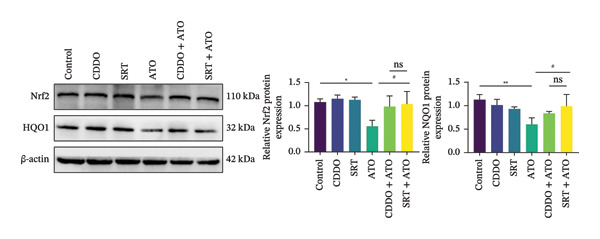
(c)

**Figure:**
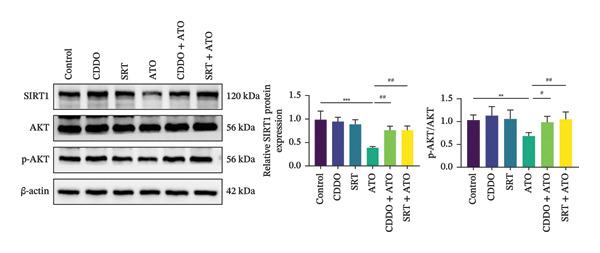
(d)

## 4. Discussion

**FIGURE 10:**
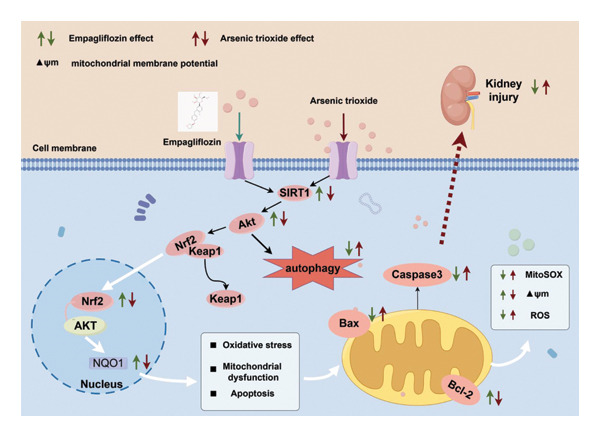
Diagram of the mechanism of EMPA inhibiting ATO‐induced nephrotoxicity by regulating the SIRT1/Akt/Nrf2 signaling pathway.

ATO is used to treat newly diagnosed and relapsed APL patients; however, adverse reactions such as nephrotoxicity, cardiotoxicity, and edema during clinical treatment limit its clinical application [25]. The kidney plays a crucial role as a target organ in terms of arsenic toxicity and accumulation. We aim to find a drug with sufficient safety to prevent ATO‐induced nephrotoxicity. EMPA was originally used to treat Type 2 diabetes in clinical practice, and it has been found to significantly reduce the risk of new or worsening kidney disease by 39% in clinical applications [26]. This study is the first to confirm the protective effect of EMPA against ATO‐induced nephrotoxicity. As shown in Figure 10, we found that EMPA can alleviate ATO‐induced kidney damage, oxidative stress, apoptosis, and the decline in MMP. This protective effect may be partially achieved through the activation of the SIRT1/Akt/Nrf2 signaling pathway.

The evaluation of organ coefficients in toxicological research is crucial for identifying potentially harmful effects of chemicals. Serum levels of BUN and CREA are widely regarded as the primary indicators of renal function in clinical settings. In the present study, the ratio of left kidney weight to body weight in mice exposed to ATO increased and exhibited significant renal structural damage, along with elevated serum BUN and CRE levels. EMPA alleviated renal pathological changes, including inflammatory cell infiltration, tubular epithelial edema, and necrosis, while also improving serum BUN and CRE levels. ATO can induce cell apoptosis [27]. Bcl‐2 expression significantly decreased after ATO administration, while the expression of Caspase‐3 and Bax increased, confirming the increased apoptosis of renal cells in mice. EMPA treatment restored the expression changes of Bcl‐2, Caspase‐3, and Bax to normal levels. These findings indicate that EMPA can mitigate ATO‐induced kidney injury in mice.

Oxidative stress refers to an imbalance between pro‐oxidants and antioxidants and is a significant contributor to the development of numerous human diseases [28]. A considerable amount of evidence indicates that oxidative stress is crucial in the nephrotoxic effects induced by ATO [29]. The findings of our study indicated that antioxidant levels were reduced, while ROS generation was elevated in the kidneys of mice subjected to ATO exposure. Previous research has demonstrated that SGLT2 inhibitors can alleviate oxidative stress in both the heart [30] and kidneys [31], enhancing the antioxidant defense mechanisms, including SOD and GSH [32]. The presence of oxidative stress contributes to lipid peroxidation, which in turn causes an overproduction of MDA. EMPA significantly increased serum antioxidant enzyme levels in mice, including SOD and GSH, while inhibiting the production of MDA and ROS (Figure 2). We also conducted in vitro experiments on HEK293T cells to investigate the relationship between EMPA and ATO toxicity. We found a significant decrease in cell viability and an increase in apoptosis following ATO treatment (Figure 4). Additionally, elevated oxidative stress levels and increased ROS generation were detected in HEK293T cells treated with ATO. In cells treated with EMPA, all these changes were alleviated (Figure 5). These findings are consistent with the in vivo experiments.

Mitochondria are regarded as the main origin of cellular ROS. Damage to these organelles may result in increased ROS production, and elevated ROS levels can induce oxidative stress, changes in mitochondrial dynamics, and ultimately trigger cell apoptosis [33]. Studies have demonstrated that ATO, recognized as a clastogenic agent, is capable of causing DNA damage and mutations through modifications in MMP, in addition to inducing mitotic arrest and apoptosis [34]. Therefore, exploring pharmaceuticals that can directly or indirectly safeguard mitochondrial integrity, regulate and sustain mitochondrial homeostasis, and improve mitochondrial function is likely to emerge as a crucial approach in preventing and treating ATO‐induced nephrotoxicity. Several studies have reported a link between SGLT2 inhibitors and mitochondrial function. EMPA has been shown to improve mitochondrial function in heart failure induced by pressure overload [35]. Mitochondrial dysfunction may follow mitochondrial structural disorder, leading to a decrease in MMP and an increase in the production of mitochondrial ROS [36]. Therefore, in this study, we validated the effects of ATO and EMPA on renal mitochondrial function at the cellular level (Figure 5). The results indicate that ATO stimulation disrupts mitochondrial structure and function. EMPA treatment reduced the amount of mitochondrial superoxide anions induced by ATO, restoring MMP to normal levels.

SIRT1 plays a significant role in numerous physiological and pathological processes, including aging, energy metabolism, apoptosis, and inflammatory responses, and is essential for the regulation of mitochondrial biogenesis and metabolism [37]. Moreover, current evidence suggests that SGLT2 inhibitors exert protective effects in heart failure by upregulating SIRT1 and its downstream effectors [38]. In both in vitro and in vivo experiments, SIRT1 was significantly reduced in the ATO treatment group. Akt is a key mediator of cell survival, and Akt‐mediated Nrf2 transcriptional activation can decrease mitochondrial ROS levels [39]. SIRT1 primarily regulates Akt activity, localization, and downstream signaling through its deacetylation function. Specific lysine residues (e.g., Lys14, Lys20) within the Akt protein, particularly in its PH domain, are subject to acetylation. SIRT1 can directly bind to and deacetylate Akt. Acetylation of Akt, especially at Lys14 and Lys20, impedes the interaction between its PH domain and membrane‐bound PIP3, thereby inhibiting the recruitment of Akt to the plasma membrane—a crucial step for its full activation by PDK1 and mTORC2. Conversely, SIRT1‐mediated deacetylation facilitates Akt membrane translocation [40]. The findings of this study indicate that ATO inhibited the expression of *p*‐Akt and Nrf2 both in vitro and in vivo. In contrast, EMPA treatment was able to restore the suppression of the SIRT1/Akt/Nrf2 pathway caused by ATO and upregulate the expression of the Nrf2 downstream target NQO1 (Figures 3 and 7). To verify the role of the SIRT1/Akt/Nrf2 pathway in ATO‐induced renal toxicity, we introduced the SIRT1 activators SRT1460 and CDDO into the experiments (Figure 8). The results showed that both SRT1460 and CDDO alleviated the suppression of the SIRT1/Akt/Nrf2 pathway and its downstream NQO1 induced by ATO, protecting mitochondrial morphology and function. The above results indicate that activating the SIRT1/Akt/Nrf2 signaling pathway can counteract mitochondrial damage induced by ATO.

Autophagy is a highly conserved intracellular degradation system found in eukaryotic organisms. This process involves the encapsulation of damaged cytoplasmic organelles, proteins, and macromolecules by double‐membrane structures known as autophagosomes, which are then transported to lysosomes for degradation, facilitating the recycling of the resulting products [41]. Furthermore, autophagy is widely recognized as a mechanism that promotes cell survival, largely due to the significant link between its dysregulation and nonapoptotic forms of cell death [42]. There exist basal levels of autophagy within cells in order to protect cells against various extracellular stresses, such as starvation or hypoxia [43]. ATO stimulates autophagy through the modulation of the PI3K/Akt/mTOR signaling pathway, which in turn encourages apoptosis in cancer cells [44]. We examined the impact of autophagy on the nephrotoxic effects induced by ATO. The results indicate that ATO promotes autophagy both in vivo and in vitro. A low dose of the autophagy inhibitor 3‐MA can alleviate the decrease in cell viability induced by ATO (Figure 4(c)).

SIRT1 plays a key role in promoting autophagy through its regulatory network, and in this study, we found that ATO can enhance autophagy in mouse kidneys and HEK293T cells while suppressing SIRT1 expression. When evaluating the effects of ATO and EMPA on SIRT1 mRNA expression in HEK293T cells, the results showed that ATO did not significantly inhibit SIRT1 mRNA levels, but its protein expression was significantly reduced, suggesting that this phenomenon may not be related to mRNA expression or stability. Autophagy is a lysosome‐dependent self‐degradation process that degrades and recycles damaged organelles and proteins through the autophagosome–lysosome pathway. Recent studies have indicated that excessive activation of autophagy can mediate the degradation of SIRT1 protein during mammalian aging [24]. To clarify the molecular mechanism underlying ATO‐mediated SIRT1 regulation, this investigation employed the autophagosome–lysosomal inhibitor Lys05 in co‐treatment with ATO on HEK293T cells. Experimental data demonstrated that Lys05 administration substantially rescued ATO‐induced SIRT1 protein depletion. These findings collectively suggest that during nephrotoxic progression, ATO induces proteolytic degradation of SIRT1 through autophagosome–lysosomal machinery hyperactivation, thereby mechanistically explaining the observed discrepancy between SIRT1 protein depletion and stable mRNA expression levels under ATO exposure.

Numerous research efforts examining the influence of SGLT2 inhibitors on autophagy suggest that these inhibitors have the potential to stimulate stalled autophagic flux. However, some studies propose that SGLT2 inhibitors may either activate or inhibit autophagy flux, contingent upon specific conditions [45]. Interestingly, EMPA inhibits the expression of LC3‐II and increases the level of P62 protein, suggesting that EMPA can suppress ATO‐induced autophagy (Figures 3(c), 7(e)). Based on the above findings, we tend to believe that EMPA can ameliorate the dysregulation of autophagy induced by ATO.

This study has several limitations that should be considered. While our findings confirm that EMPA alleviates ATO‐induced renal toxicity, whether EMPA directly activates SIRT1 and the relationship between mitochondrial protection and the SIRT1/Akt/Nrf2 signaling pathway require further validation. The current verification was conducted only in HEK293T cells; future studies should include additional cell lines, such as HK‐2 cells. Moreover, whether the combination of EMPA and ATO exerts synergistic effects on anticancer activity remains to be investigated in both animal models and cellular systems, such as NB4 cells.

## 5. Conclusion

In conclusion, ATO induces kidney dysfunction, oxidative stress, apoptosis, autophagy, and mitochondrial damage, while also inhibiting the SIRT1/Akt/Nrf2 signaling pathway. This study provides the first evidence of the protective effects of EMPA against ATO‐induced nephrotoxicity. EMPA alleviates ATO‐induced oxidative stress, apoptosis, and autophagy, and it safeguards mitochondrial morphology and functional integrity by activating the SIRT1/Akt/Nrf2 signaling pathway. As a potential SIRT1 activator, EMPA may offer greater clinical utility by alleviating the renal toxicity associated with ATO.

## Author Contributions

Chunrong Pang: conceptualization, data curation, methodology, formal analysis, writing–original draft, writing–review and editing. Wenlei Zhang: formal analysis, data curation, validation. Chenli Yue: formal analysis, software, validation. Haoxuan Li, Jinyan Li, Xinru Wang, and Xinsheng Duan: formal analysis, data curation. Longyu Li: validation. Zengliang Gao: validation. Xin Hai: conceptualization, supervision, writing–review and editing, funding acquisition.

## Funding

This study was supported by the National Natural Science Foundation of China (No. 82274028), the Natural Science Foundation of Heilongjiang Province (No. LH2023H032), and the Key Project of Natural Science Foundation of Heilongjiang Province (No. ZL2024H005).

## Disclosure

All authors contributed to the article and approved the submitted version.

## Ethics Statement

The authors assert that all procedures contributing to this work comply with the ethical standards of the relevant national and institutional committees on human experimentation and with the Helsinki Declaration of 1975, as revised in 2008. The authors assert that all procedures contributing to this work comply with the ethical standards of the relevant national and institutional guides on the care and use of laboratory animals. This study is not a clinical trial. Clinical trial number: not applicable.

All animal experiments, data description, and statistical analyses complied with the ARRIVE guidelines.

## Conflicts of Interest

The authors declare no conflicts of interest.

## Data Availability

The data that support the findings of this study are available from the corresponding author upon request. The data are not publicly available due to privacy or ethical restrictions.
